# PAI-1 and CHRNA1—A new molecular axis and therapeutic target in primary focal hyperhidrosis

**DOI:** 10.1016/j.omtn.2025.102664

**Published:** 2025-08-15

**Authors:** Marie Hoareau, Rong-Mo Zhang

**Affiliations:** 1 Yale Cardiovascular Research Center, Section of Cardiovascular Medicine, Department of Internal Medicine, Yale School of Medicine, 300 George Street, New Haven, CT 06511, USA; 2 Yale Stem Cell Center, 10 Amistad Street, New Haven, CT 06511, USA

## Main text

Primary focal hyperhidrosis (PFH, OMIM no. 144110), affecting 1%–6.1% of the population,^1^ is characterized by excessive and localized sweating, typically on the palms, soles, underarms, face, or scalp. Current treatments focus largely on topical blocking of sweating, although other strategies exist, such as local treatments (iontophoresis and intradermal injections), systemic medication, and local surgical procedures to remove the sweat glands.^2^ While these approaches are effective, they are symptomatic, urging a mechanical understanding of PFH pathogenesis and the development of better adapted therapeutic options. The recent study by Zheng et al.^3^ describes a new player and potential therapeutic target: the plasminogen activator inhibitor-1 (PAI-1), which was found to be decreased in both patients with PFH and in a hyperhidrosis mouse model. These findings offer a new perspective on PFH pathophysiology and therapy by linking PAI-1 to regulation of the nicotinic acetylcholine receptor alpha 1 subunit (CHRNA1), a key player in sweat gland function.

**Figure 1:**
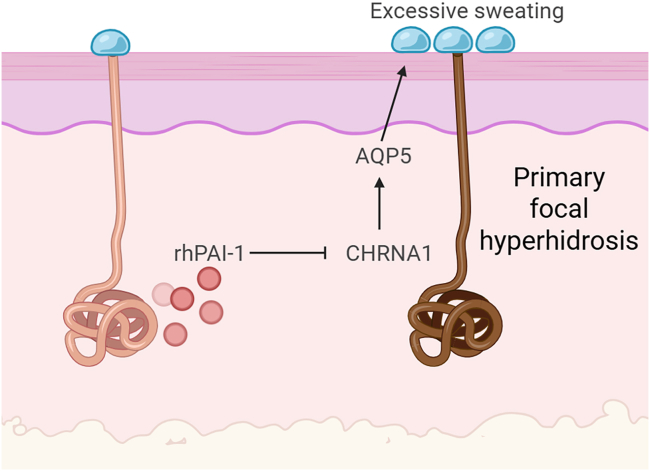
rhPAI-1 alleviates primary focal hyperhidrosis by downregulating CHRNA1, which leads to reduced AQP5 expression and decreased sweat secretion

In their study, Zheng et al. analyzed sweat gland tissues from patients with PFH, revealing that patients with PFH exhibited significantly reduced PAI-1 expression and elevated CHRNA1 expression at both the mRNA and protein levels, as compared to healthy controls. This negative correlation holds true across different PFH subtypes (axillary, craniofacial, and palmar) and in a pilocarpine-induced hyperhidrosis mouse model. These results were further validated in isolated primary sweat gland cells, mainly epithelial cells from patients, confirming that the PAI-1/CHRNA1 relationship is intrinsic to the glandular tissue itself. Treatment with recombinant human PAI-1 (rhPAI-1) to sweat gland cells downregulated CHRNA1 and aquaporin 5 (AQP5)—the latter being a crucial water channel for sweat secretion (Figure 1). *In vivo*, rhPAI-1 administration led to a dose-dependent reduction in sweat secretion and serum acetylcholine levels, supporting the hypothesis that PAI-1 acts as a molecular brake on cholinergic signaling and sweat production. This result is in line with their previous *Pai1* knockout mice, which show an exacerbated response to the pilocarpine challenge used to induce hyperhidrosis, as well as an increased level of CHRNA1 compared to wild-type mice challenged the same way.^4^

PFH has been described as a disorder related to a dysfunctional regulation of sweat production. Some modifications involving the autonomous nervous system proximally to the sweat glands have been described in patients at different levels: sympathetic ganglia, neurons, and more locally in the sweat gland tissue.^2^ At the glandular level, excessive sweating requires the neurophysiological interactions of multiple cell types, including clear cells, myoepithelial cells, and dark cells, all stimulated by acetylcholine from the sympathetic nerve fibers responding to thermal and emotional stimuli.^5^ Current and previous studies^3^^,^^4^ show that both restoring PAI-1 and inhibiting CHRNA1, an acetylcholine receptor, could ameliorate PFH. This inhibitory effect of PAI-1 on CHRNA1 was elucidated *in vitro*, using isolated epithelial cells from sweat glands. As the major function of myoepithelial cells is to facilitate sweating produced from secretory cells via contraction induced by acetylcholine,^6^ it would be interesting to see whether some contractile markers, such as α-smooth muscle actin or myosin heavy chains, are decreased by PAI-1. Other than myoepithelial cells, the secretory cells, namely clear and dark cells, are the major producers of water and other components in sweat. In these cells, the water channel proteins, like AQP5, are critical for its function in PFH. Thus, whether the inhibitory effect of PAI-1 on AQP5 is also applicable to secretory cells would be intriguing for us to understand the molecular mechanism of PFH pathogenesis. Further studies could focus on dissecting the functions of PAI-1/CHRNA1 axis in both myoepithelial cells and secretory cells, by employing single-cell RNA sequencing analyses and cell-specific genetic knockout models. Clarifying these relationships will guide the development of more precise interventions.

Beyond its role in the CHRNA1/AQP5 signaling axis, PAI-1 is known to be a major modulator of the extracellular matrix (ECM) by regulating matrix metalloproteinases.^7^ The major ECM component around sweat glands is the basement membrane,^8^ composed mainly of collagens and proteoglycans. These ECM proteins provide mechanical support by directly interacting with the myoepithelium, which is critical for myoepithelial cell maturation and contraction. Furthermore, the ECM serves as a reservoir of multiple growth factors and space for neurotransmitters regulating both sweat production and secretion.^9^ Therefore, characterizing the biochemistry and mechanic changes in the ECM in PFH and investigating whether and how changes in PAI-1 alter the basement membrane around sweat glands will provide molecular and therapeutic insights on PFH pathogenesis.

The phenotypic outcome in the PFH mouse model is promising, but as with any protein therapy, more studies on the immunogenicity and pharmacokinetics of rhPAI-1 injection are required. The frequency of administration of the treatment could also be optimized, as daily injections of a recombinant protein might not be the most convenient strategy for clinical applications. PAI-1 also seems to act on PFH primarily via CHRNA1-dependent pathways, suggesting CHRNA1 could be a more direct and efficient therapeutic target. Additionally, it is worth noting that elevated PAI-1 is associated with increased risk of thrombosis, fibrosis, vascular aging, and metabolic syndrome.^10^ Most therapeutic studies in cardiovascular and fibrotic diseases have focused on inhibiting PAI-1, not enhancing it. Systemic administration of PAI-1, as performed in Zheng et al.’s study, could therefore carry risks to patients with underlying cardiovascular or metabolic vulnerabilities. Thus, future studies should rigorously assess the side effects of rhPAI-1 injection, particularly in the cardiovascular and skin systems.

Overall, PFH remains an understudied disorder, whose complexity stems from the involvement of multiple cell types, including gland cells (myoepithelial cells, clear and dark cells), and neurons. Attempts to identify genetic causes are largely hindered by the genetic heterogeneity of PFH,^1^ further suggesting that multiple cellular and non-cellular factors are involved. In their article, Zheng et al. identify PAI-1 as a key inhibitor of CHRNA1 hyperactivity in sweat gland epithelial cells, shedding new light on the molecular mechanisms involved in PFH pathogenesis. Though this negative correlation is robust, the precise regulatory mechanism between them needs to be further clarified. Non-cellular factors regulating crosstalk between all the cells involved in PFH, including neurotransmitters and ECM, need to be further characterized.

In conclusion, Zheng et al. raised multiple intriguing aspects of PFH, including crosstalk between PAI-1 and CHRNA1, gland cell type contribution, and secondary effects of injected rhPAI-1, among others. Investigating the underlying molecular mechanism aspects is now essential to enhance our understanding of PFH pathogenesis and provide, in the future, better and more targeted treatments for patients suffering from PFH.

## Declaration of interests

The authors declare no competing interests.
